# Microbial Effectors: Key Determinants in Plant Health and Disease

**DOI:** 10.3390/microorganisms10101980

**Published:** 2022-10-06

**Authors:** Jewel Nicole Anna Todd, Karla Gisel Carreón-Anguiano, Ignacio Islas-Flores, Blondy Canto-Canché

**Affiliations:** 1 Unidad de Biotecnología, Centro de Investigación Científica de Yucatán, A.C., Calle 43 No. 130 x 32 y 34, Colonia Chuburná de Hidalgo, Mérida C.P. 97205, Yucatán, Mexico; 2 Unidad de Bioquímica y Biología Molecular de Plantas, Centro de Investigación Científica de Yucatán, A.C., Calle 43 No. 130 x 32 y 34, Colonia Chuburná de Hidalgo, Mérida C.P. 97205, Yucatán, Mexico

**Keywords:** microbial effectors, effectoromics, effectors in plant health, effectors in plant disease

## Abstract

Effectors are small, secreted molecules that alter host cell structure and function, thereby facilitating infection or triggering a defense response. Effectoromics studies have focused on effectors in plant–pathogen interactions, where their contributions to virulence are determined in the plant host, i.e., whether the effector induces resistance or susceptibility to plant disease. Effector molecules from plant pathogenic microorganisms such as fungi, oomycetes and bacteria are major disease determinants. Interestingly, the effectors of non-pathogenic plant organisms such as endophytes display similar functions but have different outcomes for plant health. Endophyte effectors commonly aid in the establishment of mutualistic interactions with the plant and contribute to plant health through the induction of systemic resistance against pathogens, while pathogenic effectors mainly debilitate the plant’s immune response, resulting in the establishment of disease. Effectors of plant pathogens as well as plant endophytes are tools to be considered in effectoromics for the development of novel strategies for disease management. This review aims to present effectors in their roles as promotors of health or disease for the plant host.

## 1. Introduction

The survival of organisms in their respective environments can be attributed to genome evolution and natural selection that have propagated and maintained the genes necessary for them to thrive. An important subset of these genes encodes molecules called effectors. They are traditionally defined as pathogen proteins that alter host cell structure and physiology, thereby facilitating infection or inducing a defense response [1,2]. Effectors have since been discovered in non-pathogenic organisms such as mycorrhizae and rhizobacteria, cementing their place as essential molecules across ecological interactions with the plant host. We define effectors as secreted or translocated molecules that influence organisms’ interactions with each other, usually to the benefit of the producer organism. These molecules induce physical and physiological changes in other organisms, and in some cases, in the said producer organism, influencing their interaction with others. These molecules can be proteins [3,4,5], secondary metabolites [6,7,8] or small RNAs [9,10,11], but the majority of characterized effectors are proteins [12,13].

Effector molecules are involved in microbe penetration and proliferation in the host, suppression of host immune responses and nutrient acquisition [14,15,16,17], and though these genes are encoded in the genome of an organism, the secreted or translocated gene products mainly function in the plant host [4,18]. Effector molecules are integral to plant-microbe interactions, having been identified in insects [19,20], nematodes [21,22,23], fungi and oomycetes [24,25,26], bacteria [27,28], viruses [29,30] and, surprisingly, in plants [31].

Effectors allow the pathogen or endophyte to colonize the plant host through various mechanisms; these include preventing recognition by the host, regulating host gene expression, interfering with phytohormone defense pathways and influencing host protein trafficking [15,32,33,34]. Common targets of effectors of bacteria, oomycetes and fungi include host proteases, the ubiquitin-proteasome system, autophagy components, reactive oxygen species (ROS) homeostasis, immune receptors and phytohormones [13,35,36]. Once the effector leaves the producer organism, it may target the host apoplast [37,38] or the cytoplasm, where many effectors target intracellular organelles [39,40,41]. Fluorescence protein-tagging coupled with confocal microscopy and protein–protein interaction experiments such as co-immunoprecipitation and yeast-2-hybrid assays, are techniques that have been commonly used to identify effector targets in model plants amenable to transformation. Increasing reports show effectors at the helm of pathogen invasions, where they induce disease susceptibility, although the majority of effector targets remain unknown [18,26].

Many effector targets are associated with plant protection and are positive regulators of plant immunity, such as the NBS-LRR receptors that are often resistance (R) gene products. Other targets are associated with susceptibility, plant genes that foster the establishment of an infection and, as such, are negative regulators of plant immunity [26,42,43]. With respect to these susceptibility factors, their overexpression in the host results in increased pathogen growth and their deletion results in a reduction in disease symptomology or loss of susceptibility [44,45]. We are still understanding the interactions between receptors and effectors; the interaction between susceptibility factors and effectors is one that is more challenging to understand [46]. The exploitation of these targets was once heavily associated with necrotrophic pathogens during host colonization, but recent investigations have highlighted biotrophic pathogen effectors targeting host susceptibility (S) gene products [45,47,48].

The identification of effectors in phytopathogens has dominated effectoromics. Understanding their effectors and their interactions with host targets is important for safeguarding plant health. Effector-based screening of germplasm containing resistance genes has been useful for resistance breeding [49,50] and finding novel resistance genes [51,52]. Concurrently, effector-assisted selection of plants lacking susceptibility genes or selection of plants with reduced sensitivity to certain susceptibility-targeting effectors is also occurring [53,54]. Likewise, the mutation of these susceptibility genes in plants may confer a more durable resistance than that which is mediated by resistance genes [55,56,57], and successful S gene mutation using CRISPR gene editing was recently documented in rice for resistance against the bacterial blight causal agent, *Xanthomonas oryzae* pv. oryzae [58]. Unfortunately, effectors are constantly evolving to outwit their hosts, putting plant health at risk; effector-triggered defense or R-gene mediated resistance is constantly being overcome by the crafty pathogens in their pathosystems [59,60,61,62]. On the other hand, effectors in plant beneficial organisms are lesser-studied molecules in effectoromics but represent a mine of underdeveloped potential for disease management. This review aims to highlight some of the interesting effectors recently identified in plant-pathogenic and mutualistic microorganisms and forges a path for better effector identification and implementation in plant protection

## 2. Effectors and Plant Defense

In order to better understand how effectors function, the role of effectors in plant immunity is discussed. Plants have an innate immune system comprised of two levels: MAMP-triggered immunity (MTI) and effector-triggered immunity (ETI). We only briefly discuss these concepts here as they have been amply discussed elsewhere [63,64,65,66,67]. In MTI, microbe-associated molecular patterns, or MAMPs, are defined as broadly conserved molecules common to various organisms, e.g., fungal chitin and bacterial flagellin. MAMPs are commonly recognized by transmembrane pattern recognition receptors (PRRs) in the plant apoplast. Effectors, on the other hand, have conceptually been understood as less-conserved molecules (although exceptions exist where effectors are highly conserved among varying species [68,69]).

Our understanding of effectors, in large part, is owed to the investigation of plant–pathogen interactions. Effectors were first called ‘avirulence factors’ by the botanist Flor in the 1940s [70]; these proteins (shortened to ‘Avr’) are recognized in the plant by a cognate ‘R’ or resistance protein, which confers resistance to that particular pathogen. Resistance proteins are receptors mostly belonging to the family of nucleotide binding (NB) and leucine rich repeat (LRR) domain (NB-LRR) proteins [71,72]. The recognition of the pathogen Avr protein by the resistance protein R results in an incompatible interaction, producing an inhospitable environment for the pathogen which stymies disease progression. This Avr–R interaction was dubbed the ‘gene-for-gene hypothesis’ and in Flor’s work it was applied to the fungus *Melampsora lini* and the plant host, the flax plant, *Linum usitatissimum*. The name ‘effectors’ was more aptly adopted later, as the molecule could display virulent or avirulent activity, depending on whether the host possesses the resistance gene or not and can therefore have a positive or negative effect on the fitness of the pathogen and its ability to cause disease [2,73]. Avrs, by definition, are effectors that trigger ETI resulting in the visible dry necrotic lesions of the hypersensitive response. Resistance genes of the host are molecular land mines; once they are tripped by the pathogen Avr, the plant launches a defense response against the invading organism (ETI), which is disadvantageous to the pathogen. Since the effector is expected to benefit the producer organism, it is suspected that each Avr protein has a primary function in virulence, but this activity is masked when the effector is recognized by the dominant resistance protein of the plant [74].

**Figure 1:**
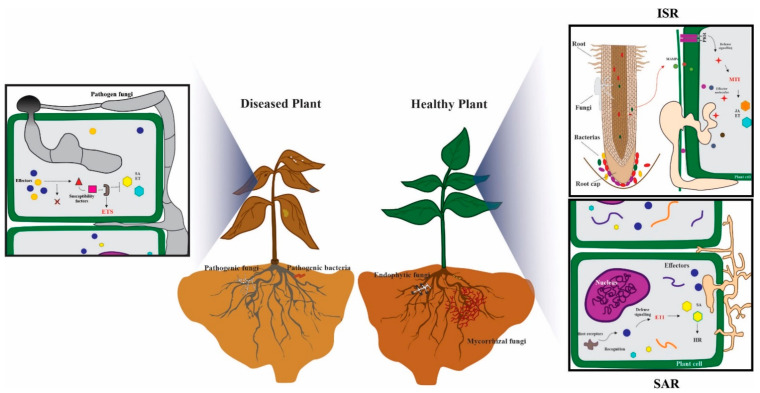
Effectors are major determinants of plant disease. Pathogens secrete effectors that can induce disease susceptibility through targeting host susceptibility factors and subverting the ETI defense response resulting in disease, ETS, (**left**). On the other hand, pathogens can also induce ETI and systemic acquired resistance (SAR) when hosts have the corresponding resistance proteins that recognize Avr effectors, producing the HR and stopping the progression of the pathogen (**right**). Conserved molecules, called MAMPs, from both pathogens and mutualists elicit the MTI defense response in the host; mutualist MAMPs induce ISR. Endophytic fungi and rhizobacteria as well as mycorrhizae promote plant defense through the activation of ISR and the plant becomes “primed” to resist infection from incoming pathogens (**right**).

MAMPs and effectors are both elicitors of host defense mechanisms. MTI and ETI are described as “stages” in a model described for plant immunity called the zig-zig model [63]. In MTI, the MAMPs of the pathogen are recognized by plant receptors resulting in the deposition of callose, induction of mitogen-activated kinase (MAPK) signaling, induction of pathogenicity related proteins and the oxidative burst (production of reactive oxygen species or ROS) in the plant host [75]. MTI culminates with the pathogen being unable to progress with the infection and the plants remaining healthy. Pathogens release effectors to hamper this first stage of immunity (MTI) resulting in effector-triggered susceptibility (ETS). Plants, in turn, have evolved with receptors (resistance proteins) which recognize these (Avr) effector molecules and trigger effector-triggered immunity or ETI, a hallmark of the incompatible plant–pathogen interaction previously mentioned. ETI is characterized by an oxidative burst and the upregulation of defense-related proteins such as phytoalexins and can culminate in the hypersensitive response (HR) (a type of programmed cell death or PCD) that ultimately stops pathogen growth at the site of infection. In the last stage of the zig-zag model, the pathogens outwit the plants once again; they evade recognition by the plant’s receptors by modifying the effector genes or using other effectors that help suppress the ETI response in the plant and target plant proteins (Figure 1). Recently, great advances have been made in the understanding of plant immunity. It is now better appreciated that MTI and ETI are not static stages in plant defense, but rather an interconnected system where one relies on the other [66,76].

### 2.1. Effectors in Plant–Pathogen Interactions

Although the original discovery of Flor’s gene-for-gene hypothesis was made between the flax plant and the rust fungus, it has since been demonstrated that effectors are key to all plant–pathogen interactions. The Avr effector, commonly associated with pathogenesis, is only one type of effector among various that can influence organisms’ interactions. Effectors of the major disease-causing organisms that compromise plant health and their roles in the establishment of plant-pathogen interactions are presented.

### 2.2. Fungal and Oomycete Effectors

Fungal and oomycete pathogens are the major disease-causing eukaryotic microorganisms [77,78]. They were once believed to be in the same kingdom due to their similar morphologies and lifestyles but have since been separated into different kingdoms as fungi (kingdom: Fungi) are evolutionarily more similar to animals, and the oomycetes (kingdom: Chromista) to golden-brown algae. These organisms are similar in their vegetative growth phase, where they both produce thread-like mycelia and form sexual and asexual spores. They are also similar in their production of haustoria-specialized feeding appendages that form an interface between them and the plant cell to retrieve plant nutrients and to release effector molecules [79].

Fungi and oomycetes present three main lifestyles: biotrophy, hemibiotrophy and necrotrophy. Biotrophic organisms require live hosts to complete their life cycle, and their effectors allow them to stealthily enter and remain in the host while avoiding recognition and suppressing the host’s defenses to maintain an optimum environment. Many effectors prevent MTI from being induced in the plant host such as Foa3 of *Fusarium oxysporum* [80] and Rip1 of *Ustilago maydis* [81]. Some effectors help in the establishment of pathogen reproductive structures such as BAS2 of *Colletotrichum gloeosporioides* [82], while others aid hyphal attachment and proliferation in the host, such as lep1 of *Ustilago maydis* [83]. This is just the tip of the iceberg for fungal effector functions, and comprehensive reviews on fungal and oomycete effector functions can be found elsewhere [18,26,35,84].

While biotrophic effectors often suppress host immunity and generally avoid setting off alarms in the plant host, necrotrophic fungi such as *Sclerotinia sclerotium* and *Botrytis cinerea* have a more aggressive approach and induce cell death in susceptible hosts with the help of their effectors. Hemibiotrophic pathogens such as the oomycete *Phytophthora infestans* employ both mechanisms; they suppress cell death early in the biotrophic phase, but, at later stages, cell death-inducing effectors are upregulated. This later induction produces the necrotic tissue necessary for the pathogen to complete its disease cycle [17,32]. Hemibiotrophic fungi and oomycetes are estimated to have the largest arsenal of effectors [85]. Necrotrophs, however, have smaller effectoromes that are just as important for necrotroph pathogenicity [85,86,87]. Host-specific necrotrophs, such as wheat pathogens *Parastagonospora nodorum* and *Pyrenophora tritici-repentis,* produce effectors that interact with dominant host proteins encoded by susceptibility (S) genes. This interaction is called the inverse gene-for-gene interaction because the interaction between necrotrophic host specific toxins (HST) and the S protein results in susceptibility (disease) instead of resistance to the plant disease (health). The interaction has great similarity to the resistance protein (R) and Avr interaction, leading to an oxidative burst and programmed cell death (PCD) [88]. Victorin, a non-ribosomal peptide from *Cochliobolus victoriae* [89] and SnTox1 of *Parastagonospora nodorum* [90], is an example of a necrotrophic effector that induces PCD upon interaction with the S gene products in their hosts, inducing disease susceptibility. Examples of functionally characterized avirulence effectors (Avrs) associated with disease resistance and other effectors that are associated with susceptibility are given in Table 1.

### 2.3. Bacterial Effectors

In bacteria, effectors are commonly secreted through type III, IV and VI systems, with the type III effectors (T3Es) being the most common effector type studied [129,130]; bacteria employ a nanosyringe forming a conduit for the direct delivery of proteins to the host [131]. The roles of bacterial effectors in plant disease have been better understood thanks to functional genomics studies in the hemibiotrophic model organism, *Pseudomonas syringae* [132,133]. Many T3Es display functional redundancy which complicates studying their contribution to pathogen virulence in *P. syringae* [134].

Regarding effector types, some are defense-related avirulence effectors that induce ETI in the presence of a resistance protein, e.g., RipB [103] and RipJ [104], while other bacterial T3Es can suppress ETI and PTI-associated cell death caused by other effectors and elicitors. For example, AvrRpt2 suppresses ETI cell death caused by the effector HopA1, and HopF2 suppresses flagellin-induced PTI [135]. Similarly, RipAC suppresses ETI induced by the Avr effector RipAA [136]. The effector AvrPtoB is both a cell death inducer and suppressor. This effector can promote cell death in tomato plants which carry the Pto resistance protein but is a general cell death suppressor in *N. benthamiana* of the Cf9-mediated and Bax-mediated cell death responses as well as Pto-AvrPto-mediated cell death [134,137,138].

Host defense suppression is the main function associated with bacterial T3Es, although they also function in nutrient acquisition [139,140] and bacterial colonization and dissemination within the host [141,142]. The molecular mechanisms of these effectors include interfering with signal transduction, transcription, and host secretory pathways [13,143,144]. Perhaps one of the most fascinating classes of T3SS effectors is the transcription activator-like effectors (TALEs) found in *Xanthomonas* sp. These effectors act like transcription factors, binding to sequences in or near promoter regions of host genes and activating their transcription in the host nucleus. Targets of TALEs in the plant host include nutrient transporters [142,145] as well as various plant transcription factors involved in promoting disease susceptibility [146,147]. Predictive tools are now available for the identification of bacterial effectors in the three types of secretion systems; type III secreted effectors [148], type IV secreted effectors [149], type VI secreted effectors [150] and the improved ability to predict new effectors is driving bacterial effectoromics research.

### 2.4. Effectors in Plant-Beneficial Microbe Interactions

Two decades ago, a mycorrhizae-plant interaction first produced evidence of effectors secreted by beneficial organisms [151]. Beneficial organisms (or mutualists), as the name suggests, provide benefits to the plant host, usually by increasing nutrient availability for plant roots and inducing disease resistance. In exchange, plants provide protection and photosynthates [152]. Mutualists, such as pathogens, need to reprogram the plant’s immune system to prevent their detection by the plant. While colonizing plant roots, mutualist MAMPs are recognized as foreign molecules to the plant, setting off alarms and triggering a plant defense response. In response, beneficial microbes such as mycorrhizae and rhizobacteria have developed effectors to suppress the defense mechanisms of the plant during their colonization of plant roots. Initially, these organisms trigger MTI which is weaker in comparison with the plant’s response to true pathogens [153]. The plant later reaps the benefits of systemic resistance against a wide range of incoming pathogens (induced systemic resistance, ISR) after the symbiosis with the beneficial organism is established [154]. Beneficial organisms that are inducers of ISR include plant growth-bacteria of the genus *Pseudomonas* spp. and *Rhizobium* spp., among others, and fungi in the genus *Trichoderma* sp. and *Serendipita indica* [155,156]. Examples of their effectors and how they influence interactions with the plant host can be found in Table 2.

Many of the effectors discovered in beneficial organisms appear to target hormone signaling pathways in the plant host. The effector, SP7, of the endomycorrhiza *Glomus intraradices*, interacts with the transcription factor ERF19 involved in ethylene signaling. The effector alters the production of this phytohormone, which regulates the transcription of many defense-related genes [157]. MiSSP7, of the ectomycorrhiza *Laccaria bicolor*, interacts with plant repressor proteins PtJAZ5 and PtJAZ6 and prevents their degradation, which would otherwise result in the transcription of jasmonate acid (JA)-controlled genes that act in the plant’s defense [158]. Other effectors directly aid in the establishment of the mutualistic interaction such as RiCRN1, a Crinkler effector of *Rhizophagus irregularis* that aids in the formation of the interaction structures of the fungi called arbuscules [159]. Another *R. irregularis* effector, RiNLE1, promotes colonization by interacting with a host histone protein (H2B), preventing its ubiquitination and leading to the downregulation of defense-related genes [160].

Effectors are also key in the establishment of plant endophytic microbial communities and the interactions between endophytes and plants. Endophytes colonize plant tissues of the phyllosphere or rhizosphere, without causing apparent harm to their host and both plant and endophyte benefit from the association [161]. Mutualistic mycorrhizae are not characterized as endophytes, being phylogenetically distinct from most other endophytes [162]; root endophytes also do not commonly establish nutrient transfer interfaces like mycorrhizae do [163]. Like their mycorrhizal counterparts, endophytic organisms produce effectors that manipulate host defense, especially MTI, in order to establish the endophyte–host symbiosis; for example, the endophytic fungus *Pestalotiopsis* sp. secretes an effector with chitin deacetylase activity that hydrolyzes elicitors and chitin oligomers to prevent chitin-triggered immunity in the rice host [164]. The effector FGBI of the endophytic fungus *Piriformospora indica* is another suppressor of MTI, which prevents β-glucan-triggered immunity in the host, through its binding to β-glucans in the fungal cell wall [165].

In the best of times, endophytes are defenders of plant health in the face of biotic and abiotic stress. Endophyte-mediated resistance against plant pathogens is generated in plant hosts as a result of endophyte antagonism against pathogens [166,167,168], increased nutrient availability to the host [169,170], endophyte-produced antimicrobial compounds [171,172] and the induction of plant-produced defense compounds through effectors that modulate phytohormone pathways involved in ISR [173,174,175]. Interestingly, some endophytic effectors induce the expression of defense genes conferring protection to plants against pathogens [176] or regulate the expression of pathogen effectors to the benefit of the host [177]. Endophyte effectors associated with ISR are listed in Table 2, along with other common endophyte effector functions in plant interactions.

Endophytes are integral components of plant microbiomes, and microbial communities are indeed influenced by the endophyte-secreted molecules. Bacteria of the genera *Variovorax* and *Acidovorax*, among others, identified from the root-associated microbiome of *Arabidopsis thaliana,* were shown to protect *A. thaliana* from pathogenic fungi and oomycetes while maintaining the microbiome’s equilibrium and, in turn, plant health [178]. Interestingly, another study identified Hyde1 proteins from *Acidovorax* bacteria that were shown to have antibacterial properties against *E. coli* as well as other bacterial isolates [179], an indication that these proteins are probable effectors associated with microbial competition. A glycoside hydrolase 25 family member with lysozyme activity is another potential effector associated with microbial antagonism; this protein was found to be a major contributing factor to the antagonism of the oomycete *Albugo laibachii* by the commensal yeast *Moesziomyces bullatus* ex *Albugo* in the *A. thaliana* phyllosphere [180]. Taken together, the evidence shows that microbial incompatibility is mediated by effector molecules. Additional examples of effectors associated with microbial antagonism can be found for pathogens in competition with other microbes [181,182,183] and mycoparasites/biological control agents against pathogens [184,185].

**Figure 2:**
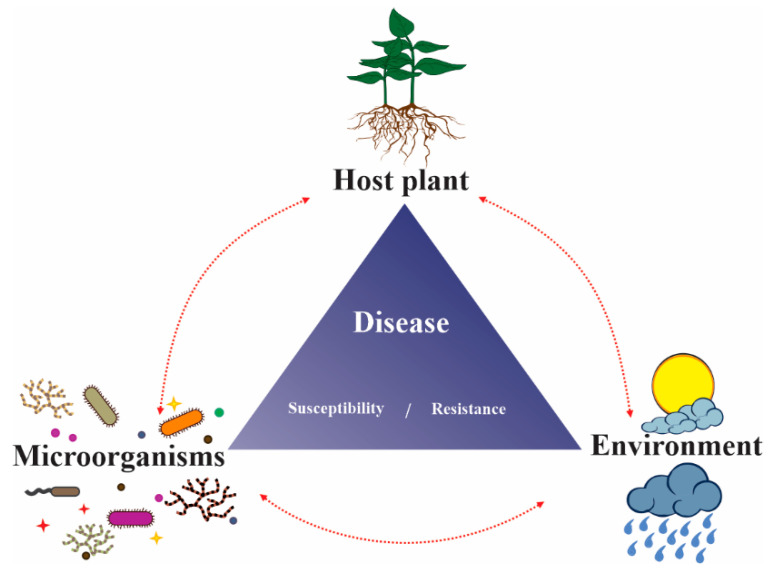
The health and disease triangle. Disease resistance or susceptibility is determined by the host plant, its microbiome and the environment. The composition of the plant microbiome is also influenced by the host plant and environmental factors. Effectors of endophytes and pathogens (left corner, represented by small circles and stars) play a major role in plant health; disease susceptibility or resistance are mediated by these molecules. Effectors are also determinants of the endophyte-pathogen lifestyle continuum and help regulate the composition of the plant microbiome. Lastly, plants regulate the environment through photosynthesis and respiration; microbes are also key components that regulate climate homeostasis.

It must be acknowledged that plant immunity is a function of innate immunity mechanisms against incoming pathogens, as well as, microbiota-mediated disease resistance [186,187]. Effectors are important determinants which shape microbial communities, determining their lifestyle, level of host specialization as well as their compatibility with other microorganisms [188]. Synergistic interactions in the plant microbiome are major contributors to plant resistance. The design and inoculation of synthetic microbial communities (SynComs) derived from native plant populations is a promising avenue for plant health promotion [178,186,189,190,191]. A noteworthy example of microbial synergism is displayed by the root endophyte *Serendipita vermifera,* which works with bacterial microbiota to confer protection against the soil-borne fungal pathogen, *Bipolaris sorokiniana* in *Arabidopsis thaliana* and barley; modulation of effector expression was observed for both the pathogenic and the endophytic fungus [192]. *A. thaliana* actively recruits and promotes the colonization of three bacterial species against infection by the pathogen, *Hyaloperonospora arabidopsidis*. In this tripartite interaction, the combination of bacteria, not any single species, significantly impacted plant protection by inducing systemic resistance in the primary plants and conferring protection to their offspring as well [193]. The plant microbiome and plant immunity are influenced by each other and are also each affected by environmental and host factors [194]; the reciprocal interplay between these components is displayed in Figure 2.

**Table 2:** Examples of effectors from beneficial microorganisms and their associated functions.

Beneficial Organism	Effector	Associated Plant	Function	References
Mycorrhizae				
*Laccaria bicolor*	MiSSP7	*Populus trichocarpa* *Populus tremula × Populus alba*	Interacts with host plant JA signaling repressors to suppress JA-related host defense signaling	[158]
*Laccaria bicolor*	MiSSP7.6	*Populus tremula x Populus alba*	Interacts with two host transcription factors: PtTrihelix1 and PtTrihelix2; involved in the establishment of Hartig net	[195]
*Laccaria bicolor*	MiSSP8	*Populus tremula x Populus alba*	Involved in mantle formation and Hartig net development for the establishment of symbiosis with host	[196]
*Glomus intraradices*	SP7	*Medicago truncatula*	Interacts with the host transcription factor ERF19 involved in ethylene-related defense signaling to suppress host defense	[157]
*Rhizophagus irregularis*	RiCRN1	*Medicago truncatula*	Localizes to plant nucleus; involved in arbuscule development	[159]
*Rhizophagus irregularis*	RiSLM	*Medicago truncatula*	Binds chitin and protects against hydrolysis by chitinases. Interferes with host chitin-triggered immunity to suppress defense response	[197]
*Pisolithus albus*	PaMiSSP10b	*Eucalyptus grandis*	Interacts with an S-adenosyl methionine decarboxylase (AdoMetDC) in the polyamine pathway; alters polyamine biosynthesis to aid colonization	[198]
*Rhizophagus irregularis*	RiNLE1	*Medicago truncatula*	Interacts with the host histone 2B protein (H2B) impairing its mono-ubiquitination which suppresses host defense-related gene expression	[160]
Endophytes				
*Bradryhizobium elkanii* USDA61	Bel2-5	*Glycine max*	Cysteine protease; involved in root nodulation	[199]
*Rhizobium* sp. NGR234	NopM	*Lablab purpureus*	E3 ubiquitin ligase; promotes root nodulation	[200]
*Rhizobium* sp. NGR234	NopE	*Glycine* *max*, *Macroptilium* *atropurpureum* and *Vigna radiata*	Calcium binding protein; regulates host root nodulation	[201]
*Serendipita indica*	FGB1	*Hordeum vulgare, Nicotiana benthamiana, Arabidopsis thaliana*	β-glucan binding lectin; alters fungal cell wall composition and suppresses β-glucan-triggered plant immunity	[165]
*Serendipita indica*	Dld1	*Hordeum vulgare*	Fungal metal ion homeostasis and micronutrient acquisition; antioxidant; enhances host root colonization	[202]
*Trichoderma asperellum*	TasXyn29.4 and TasXyn24.2	*Populus davidiana* × *P. alba* var. *pyramidalis*	Xylanases; induced Me-JA accumulation. ISR against *A. alternata*, *R. solani*, and *F. oxysporum*	[203]
*T. harzianum* Th22	Thph1 and Thph2	Maize (Inbred line Huangzao 4)	Cellulases; triggered production of (ROS) and induced genes related to the jasmonate/ethylene signaling pathway. ISR against *C. lunata*.	[173]
*T. atroviride* IMI 206040	Epl1	*Solanum lycopersicum*	Ceratoplatanin family protein; induced the expression of a host peroxidase. ISR against *A. solani* and *B. cinerea*	[204]
*T. virens* Gv29-8	Sm1	*Gossypium hirsutum*	Ceratoplatanin family protein; triggered production of ROS and induces the expression of host defense-related genes. ISR against *Colletotrichum* sp.	[205]

The endophyte–plant relationship is usually asymptomatic but sometimes endophytic microorganisms can become pathogenic due to changes in light/environment, host gene expression, nutrient balance/availability, type of host [188,206] or even an infection; a mycovirus that infected *Sclerotinia slerotorium* caused the pathogen to become an endophyte through the regulation of the expression of its pathogenicity genes [207]. The molecules which determine this continuum or the transition from one lifestyle to the other remain to be uncovered, but it is likely that effectors play a major role here. For example, in *Fusarium oxysporum*, secreted in xylem (SIX) effector profiles were different among the endophytic and pathogenic isolates assessed in the study [208]. Furthermore, a greater number of effector gene candidates and host-specific effectors were associated with pathogenicity in *F. oxysporum* compared to endophytic strains [209]. It is truly fascinating how effectors can play similar roles in pathogenic and beneficial microorganisms, but the outcomes of their interactions are contrasting. Further interactomics analyses are required to unravel these intriguing processes.

### 2.5. The New Age of Effector Identification and Characterization

The omics sciences, coupled with various bioinformatics tools, have supported investigations into the complete effector content or *effectoromes* of organisms. As such, effector identification is somewhat becoming less challenging as certain criteria have been established to identify canonical protein effector candidates. These are based on their small size (less than 400 amino acids), cysteine richness (at least 4 Cys residues, characteristic of fungal apoplastic effectors), the presence of a secretory signal peptide, the absence of transmembrane domains, overexpression data in host interactions and limited homology to proteins in other organisms [35,85,210,211]. Additionally, N-terminal effector motifs such as RXLR have been particularly important for oomycete effector identification [212,213].

Initially, it was common for researchers to establish in-house effector identification pipelines that required the use of many separate tools to determine effector candidature of a given protein. More recently, the EffHunter algorithm [85] and machine learning (ML) tools trained to predict effectors based on shared physiochemical protein properties are facilitating easier high-throughput effector identification from pathogen genomes. EffectorP versions 1, 2 and 3 (http://effectorp.csiro.au/; [214,215,216], ApoplastP v. 1.0 (http://apoplastp.csiro.au/; [217] and FunEffector-Pred (http://lab.malab.cn/~wangchao/softwares/software.html; [218] are available tools for fungi, and EffectorO for oomycetes (https://bremia.ucdavis.edu/effectorO.php; [212]; while Effectidor (https://effectidor.tau.ac.il/; [219], is a recent example of a ML predictor for T3SS effectors of bacteria.

Caution is required in the interpretation of in silico effector predictions for plant-beneficial organisms. As it becomes increasingly more evident that beneficial microorganisms also possess effectors and use their effectors in similar ways as plant pathogens, improved effector predictors should include effectors of these non-pathogenic microbes in their positive training sets to reduce the false negative rate in non-pathogenic effector identification. Saprophytes, organisms that obtain nutrients from dead or decaying organic matter, are often excluded from the effector narrative as they are considered depleted in effector molecules [214]. The possibility exists that true effectors, particularly of species in the genus *Trichoderma* sp., (well-known saprophytes that can engage in mutualistic plant interactions and in antagonism against other fungi) are being relegated to negative datasets. Predector (https://github.com/ccdmb/predector; [220], FunEffectorPred (https://github.com/ccdmb/predector, [218]) and EffectorP 3.0 (https://github.com/JanaSperschneider/EffectorP-3.0 [214]) all use secreted proteins of saprophytes that are non-pathogenic to plants to train their negative datasets. In their recent work, the authors of Predector [220], mentioned that “saprobes are not expected to possess effector proteins that facilitate plant-host infection” although they recognize that “(saprobes) may still possess proteins with similar functional or physical properties.” Undeniably, the lagging rate of characterization of effectors of beneficial microorganisms is a major limitation in the use of these proteins to train effector prediction algorithms for their use in non-pathogenic microorganisms.

## 3. Conclusions and Perspectives

The elucidation of pathogen effectoromes helps us better understand how pathogens successfully infect their hosts, causing significant crop losses in agriculture that range from food shortages to famines. In the last 20 years, our knowledge of these molecules has greatly expanded, but our understanding of effectors is still in its infancy as we continue to uncover numerous effectors and novel classes of effectors in plant-pathogenic and non-pathogenic organisms. In effectoromics, we have naturally seen a bias towards plant pathogenic effectors since these organisms are formidable threats to food security. R-gene pyramiding and S-gene manipulation through gene editing are among the prevalent effector-assisted disease control strategies [221,222,223,224]. Comparative studies are necessary to ascertain whether promoting effector-triggered defense or hindering effector-triggered susceptibility is more durable in plant protection; the suitability of each approach must be evaluated on a case-by-case basis. In the advancement of effector biology, we suggest the following lines of investigation:

(a) Bottlenecks still exist in effector identification; effectors of plant-beneficial organisms as well as those pathogenic effectors which do not possess all the canonical effector characteristics (small size, high cysteine content, etc.) may not be well represented in in silico deduced effectoromes. Newer pipelines should take these limitations into consideration, looking beyond the common physicochemical protein characteristics of effectors currently used.
(b) Effector identification is occurring at a rapid pace, but characterization is lagging relative to the large amount of effector candidates identified per organism. It is necessary to propose novel strategies and, if possible, establish standardized means of prioritizing candidates for further characterization.
(c) More attention should be placed on the effectors of plant-beneficial organisms and their characterization. This can foster effector-based screening and selection of better strains of biological control organisms for their implementation in the agricultural sector. Furthermore, the isolation and application of novel effectors from pathogens, as well as plant-beneficial organisms, may prove viable in plant protection strategies.

## Figures and Tables

**Table 1:** Examples of characterized effectors of biotrophic, necrotrophic and hemibiotrophic fungal and oomycete pathogens that are associated with disease resistance and disease susceptibility.

Effector Classification	Organism Type	Organism	Effector Name and Uniprot ID	Function	References
Resistance or Defense Associated Effectors	Biotrophic fungus	*Cladosporium fulvum*	Avr4	Induces ETI when recognized by host resistance protein Cf-4; protects fungal cell walls against hydrolysis by plant chitinases	[91,92]
	Biotrophic fungus	*Cladosporium fulvum*	Avr4E	Induces ETI; recognized by resistance protein Hcr9-4E	[93]
	Biotrophic fungus	*Cladosporium fulvum*	Ecp6	Induces ETI when recognized by resistance protein Cf-ECP6; binds to fungal chitin to prevent chitin-triggered immunity in host	[94,95]
	Biotrophic fungus	*Melamspora lini*	AvrM	Induces ETI in host; recognized by resistance protein M	[96]
	Hemibiotrophic fungus	*Magnaporthe oryzae*	AvrPia (B9WZW9)	Induces ETI in host; recognized by resistance protein RGA5	[97]
	Hemibiotrophic fungus	*Magnaporthe oryzae*	AVR-Pik (C4B8B8)	Induces ETI in host; recognized by resistance protein Pik	[98]
	Hemibiotrophic fungus	*Magnaporthe oryzae*	PWT3	Recognized by host resistance protein Rwt3	[99]
	Hemibiotrophic oomycete	*Phytophthora infestans*	AVRamr3	Recognized by host resistance protein Rpi-amr3	[100]
	Hemibiotrophic fungus	*Ascochyta lentis*	AlAvr1	Unidentified resistance gene; ETI induced in host	[101]
	Biotrophic fungus	*Puccinia polysora*	AvrRppC	Recognized by host resistance protein RppC	[102]
	Bacteria	*Ralstonia solanacearum*	RipB	Recognized by host resistance protein Roq1	[103]
	Bacteria	*Ralstonia solanacearum*	RipJ	Unidentified resistance gene; ETI induced in host	[104]
	Bacteria	*Ralstonia solanacearum*	RipAZ1	Unidentified resistance gene; ETI induced in host	[105]
	Bacteria	*Pseudomonas syringae* pv. syringae strain 61	HopA1_Pss61_	Recognized by RPS6 resistance protein; ETI induced	[106]
Susceptibility Associated Effectors	Biotrophic fungus	*Ustilago maydis*	Umrip1	Targets susceptibility factor ZmLox3, ZmLox3 represses ROS burst	[81]
	Hemibiotrophic oomycete	*Phytophthora infestans*	Pi02860	Targets susceptibility factor NRL1. NLR1 promotes degradation of positive regulator of immunity, StSWAP70	[107,108]
	Hemibiotrophic oomycete	*Phytophthora infestans*	Pi04314/RD24	Targets PP1 catalytic subunits causing their re-localization from the nucleolus to the nucleoplasm; Pi04314-PP1c holoenzymes negatively regulate salicylic acid and jasmonic acid pathways	[109]
	Hemibiotrophic oomycete	*Phytophthora sojae*	PsAvh52	Targets susceptibility factor GmTAP1, causing relocation from the cytoplasm to the nucleus. GmTAP1 promotes H3K9 acetylation to promote disease susceptibility	[110]
	Hemibiotrophic oomycete	*Phytophthora infestans*	PiAvr2	Interacts with BRI1-SUPPRESSOR1-like (BSL) BSL1, BSL2, and BSL3; BSL1 and BSL3 suppress INF1-triggered cell death (PTI)	[111,112]
	Necrotrophic fungus	*Pyrenophora tritici-repentis*	ToxA (Host-selective toxin)	Targets Tsn1, susceptibility factor involved in ToxA-triggered cell death which favors necrotrophy	[113,114]
	Necrotrophic fungus	*Parastagonospora nodorum*	SnTox1 (Host-selective toxin)	Targets Snn1, susceptibility factor involved in SnTox1-triggered cell death which favors necrotrophy; protects fungus from host chitinases	[90,115]
	Necrotophic fungus	*Pyrenophora tritici-repentis*	PtrToxB (Host-selective toxin)	Targets Tsc2, susceptibility factor involved in PtrToxB triggered cell death which favors necrotrophy	[116,117]
	Hemibiotrophic fungus	*Phytophthora sp.*	PSR2	Inhibits secondary siRNA (PPR-siRNAs) production in Arabidopsis to promote disease susceptibility	[118]
	Biotrophic oomycete	*Hyaloperonospora arabidopsidis*	HaRxL21	Responsible for transcriptional repression via interaction with TPL/TPR1 Arabidopsis proteins	[119]
	Necrotrophic fungus	*Sclerotinia sclerotiorum*	SsITL	Inhibits SA accumulation through interaction with CAS receptor in chloroplast	[120,121]
	Biotrophic fungus	*Puccinia striiformis* f. sp. *tritici*	Pst_12806	Reduces photosynthesis and ROS accumulation; interacts with TaISP, a subunit of Cyt b6/f in the chloroplast	[122]
	Biotrophic fungus	*Puccinia striiformis f. sp. tritici*	PstGSRE1	Disrupts nuclear localization of a ROS associated transcription factor TaLOL2 to suppress ROS-mediated cell death	[123]
	Biotrophic fungus	*Puccinia striiformis f. sp. tritici*	PstGSRE4	Inhibits the enzyme activity of wheat copper zinc superoxide dismutase TaCZSOD2 reducing H_2_O_2_ accumulation and HR	[124]
	Biotrophic fungus	*Puccinia striiformis f. sp. tritici*	Pst18363	Pst18363 stabilizes TaNUDX23, which suppresses ROS accumulation inducing susceptibility	[48]
	Biotrophic fungus	*Ustilaginoidea virens*	SCRE6	Interacts with and dephosphorylates the target OsMPK6 for its stabilization, suppressing plant immunity	[125]
	Bacteria	*Xanthomonas translucens pv. undulosa*	Tal8	Upregulates expression of the host gene 9-cis-epoxycarotenoid dioxygenase (TaNCED-5BS) involved in the biosynthesis of abscisic acid; decreases ex-pression of defense gene TaNPR1	[126]
	Bacteria	*Xanthomonas oryzae* pv. *oryzae*	PthXo3_JXOV_	Upregulates expression of the susceptibility gene OsSWEET14 to trigger sugar release; effector also inhibits HR and callose deposition	[127]
	Bacteria	*Ralstonia solanacearum*	RipAL	Putative lipase that catalyzes the release of linoleic acid from chloroplast lipids; induces JA production and suppresses SA signaling	[128]

ROS, reactive oxygen species; JA, jasmonic acid; SA, salicylic acid.
